# Editorial: Pulmonary fibrosis: One manifestation, various diseases

**DOI:** 10.3389/fphar.2022.1027332

**Published:** 2022-10-17

**Authors:** Barbara Ruaro, Marco Matucci Cerinic, Francesco Salton, Elisa Baratella, Marco Confalonieri, Michael Hughes

**Affiliations:** ^1^ Pulmonology Unit, Department of Medical Surgical and Health Sciences. University Hospital of Cattinara, University of Trieste, Trieste, Italy; ^2^ Unit of Immunology, Rheumatology, Department of Experimental and Clinical Medicine, IRCCS San Raffaele Hospital, University of Florence and Division of Rheumatology AOUC & Scleroderma Unit, Allergy and Rare Diseases (UnIRAR), Milan, Italy; ^3^ Department of Radiology, Department of Medicine, Surgery and Health Science, University of Trieste, Trieste, Italy; ^4^ Division of Musculoskeletal and Dermatological Sciences, Faculty of Biology, Medicine and Health, The University of Manchester & Salford Royal NHS Foundation Trust, Manchester, United Kingdom

**Keywords:** pulmonary fibrosis (PF), idiopathic pulmonary fibrosis (IPF), interstitial lung disease (ILD), familial pulmonary fibrosis (FPF), autoimmune diseases

This research topic collection entitled “**Pulmonary Fibrosis: one manifestation, various diseases** *”*, involving authors from different countries, confirms that this disease is a hot topic (Confalonieri P et al.,2022, Orlandi M et al., 2022). There are over 200 different types of pulmonary fibrosis (PF), the most common is the idiopathic pulmonary fbrosis (IPF), called idiopathic because it has no known cause. Another rare form is familial PF, for which several studies reported correlation with few genes. An important group of PF are due to other diseases, for example, autoimmune diseases such as rheumatoid arthritis, systemic sclerosis or Sjogren’s syndrome (Ruaro et al., 2022, Trombetta AC et al., 2017, Bernero Eet al., 2013). PF could correlate to viral infections (e.g. COVID-19), gastroesophageal reflux disease (GERD) (Baratella E et al, 2021, Ruaro et al., 2018), and the exposure to various materials (including naturally occurring such as bird or animal droppings, and occupational such as asbestos or silica). Furthermore, smoking, radiation treatments, and certain drugs can increase risk of developing PF. In the first article (Saketkoo et al.) of the collection, the authors evaluate the use of International Classification of Functioning, Disability, and Health (ICF) approved by World Health Organization (WHO) in patients affected by interstitial lung diseases (ILD). The results of the study supported the use of ICF in ILD, as ICF may help clinicians to collect data regarding the clinical status of their ILD patients.

The second article (Ma et al.) of the collection is an interesting and comprehensive review. The authors underlined the molecular mechanisms and pathogenic factors of IPF, which would be helpful in the diagnosis, development of new drugs and the improvement of disease prognosis. In particular, the researchers underlined the novelties regarding multiple cell types, gene mutations, epigenetic and environmental factors.

The most important message reported in the third paper (Zhou et al.) is that the assessments by high-resolution computed tomography (HRCT) pattern and scores before transbronchial cryobiopsy (TBCB) were helpful for bronchoscopists to make a better patient selection and procedure planning. The authors also reported that the multivariate analysis supported radiological probable interstitial pneumonia (UIP) pattern as an independent risk factor for moderate bleeding.

The fourth article (Zhang et al.) is a case report. The authors performed a transbronchial cryobiopsy (TBCB) assisted by extracorporeal membrane oxygenation (ECMO) in a critical case of acute respiratory failure related to an organizing pneumonia (OP) pattern. In conclusion, the paper supported that when oxygenation cannot be maintained after endotracheal intubation and surgical lung biopsy is not feasible, TBCB supported by ECMO may be a good choice to obtain lung tissue for histopathological diagnosis in patients with acute lung injury of unknown etiology.

The fifth manuscript (Zhou et al.) is an interesting case report that evaluate the treatment by pirfenidone of PF secondary to ARDS-COVID-19. Over 96 weeks after pirfenidone, the score of the mMRC dyspnea scale, the 6 min walking test distance, total lung capacity, diffusion capacity for carbon monoxide and chest CT improved. In conclusion, this case demonstrated that pirfenidone might be a potential treatment option for the post-COVID-19 pulmonary fibrosis.

The sixth article (Wang et al.) is a retrospective study that evaluate 579 patients with fibrosing ILD, of which 227 (39%) met the criteria for progression. The authors observed that clubbing of fingers and a HRCT-documented UIP-like fibrotic pattern were more frequently associated with the progressive fibrosing.

The mortality was worse in patients with PF with hypoxemia, in those with baseline diffusion capacity of the lung for carbon monoxide (DLCO)% predicted <50%, or in those with UIP-like fibrotic pattern.

In the seventh paper (Ma et al.) the researchers provides an overview of different cytokines and growth factors involved in IPF.

The authors of the eighth article (Min et al.) demonstrated that lungs from mice with bleomycin (BLM)-induced PF were characterized by decreased expression of TNF receptor-associated factor 6 (TRAF6) in lung fibroblasts. Furthermore, the results indicate that reduced TRAF6 expression in fibroblasts is essential for the progression of PF, and therefore, genetically increasing TRAF6 expression or disrupting tribbles pseudokinase 3 (TRIB3)-TRAF6 interaction could be potential therapeutic strategies for fibroproliferative lung diseases.

In the ninth article (Xu et al.) the authors used human embryonic lung fibroblasts (HELFs) treated with different concentrations of vincristine (VCR) to study the molecular mechanism of VCR-induced PF and the possible involvement of the mitogen-activated protein kinase (MAPK) signaling pathway. In the conclusions, the researchers reported that VCR could promote the differentiation of fibroblasts into myofibroblasts by regulating the MAPK signal pathway.

In the penultimate article of the collection, the authors (Tanner et al.) used a series of *in vitro* and *in vivo* models to identify the therapeutic potential of bisphosphonate zoledronic acid (ZA) in the treatment of idiopathic pulmonary fibrosis (IPF). Furthermore, farnesyl diphosphate synthase (FDPS) was used as a potential antifibrotic target using a bleomycin mouse model. The results of the study reported that *in vitro* administration of ZA reduced myofibroblast transition and blocked NF-κB signaling in macrophages leading to impaired immune cell recruitment in a transwell assay. FDPS-targeting siRNA administration significantly attenuated profibrotic cytokine production and lung damage. In addition, ZA treatment of mice with bleomycin-induced lung damage displayed decreased cytokine levels in the BALF, plasma, and lung tissue, resulting in less histologically visible fibrotic scarring. Additionally, ZA polarized macrophages towards a less profibrotic phenotype contributing to decreased IPF pathogenesis.

The last research (Yu et al.) proved that catalpol (CAT) might work through Ang Ⅱ/AT1/TGF-β/Smads pathway to improve lung pathological changes as well as suppress epithelial mesenchymal transition (EMT) in mice with PF. CAT may serve as a novel therapeutic candidate for the simultaneous blockade of Ang II and TGF-β pathway to attenuate PF.

In conclusion, this special issue pays particular attention to recently progress made on use of innovative tests and treatments, which is expected to provide new insights into research.
